# A Comparison Between Intrathecal Levobupivacaine and Bupivacaine for Quality and Safety During Infraumbilical Surgeries

**DOI:** 10.7759/cureus.30590

**Published:** 2022-10-22

**Authors:** Ayesha Goyal, Yatiraj Singi, Prashant Mallya, Ganapathi Bhat, Shankaranarayana P

**Affiliations:** 1 Cardiovascular and Thoracic Anesthesiology, All India Institute of Medical Sciences (AIIMS) Rishikesh, Rishikesh, IND; 2 Forensic Medicine and Toxicology, All India Institute of Medical Sciences (AIIMS) Bilaspur, Bilaspur, IND; 3 Anesthesiology, Kasturba Medical College (KMC) Hospital, Mangalore, IND; 4 Anesthesiology, Kurunji Venkatramana Gowda (KVG) Medical College, Sullia, IND; 5 Anesthesiology, Yenepoya Medical College, Mangalore, IND

**Keywords:** intrathecal anesthesia, intraoperative hypotension, sensory blockade, infraumbilical surgeries, levobupivacaine

## Abstract

Background

Levobupivacaine toxicity reports are rare, and when they do occur, toxic symptoms are frequently treatable with minimal morbidity and mortality. However, levobupivacaine has not entirely replaced bupivacaine in clinical practice. Moreover, the experience of intrathecal anesthesia with levobupivacaine is not well documented. Hence, the purpose of this study is to assess the quality and duration of sensory and motor blockade of levobupivacaine and its side effects, if any, compared to intrathecal bupivacaine during infraumbilical surgeries.

Methods

After approval by the Institutional Ethical Committee of Kurunji Venkatramana Gowda (KVG) Medical College and Hospital, Sullia, 90 patients aged between 18 and 65 years, of either sex, who were scheduled for elective abdominoperineal, urological, or lower limb surgeries under intrathecal anesthesia were enrolled in this prospective study from January 2013 to June 2014. The selected patients were randomly assigned to three groups of 30 each: group HB (3 mL of 0.5% hyperbaric bupivacaine), group IB (3 mL of 0.5% isobaric bupivacaine), and group IL (3 mL of 0.5% isobaric levobupivacaine). Motor blockade was assessed using the modified Bromage scale. Intergroup comparison was done using Tukey’s post hoc test. The incidence of adverse effects was analyzed using a chi-squared test. Significance was defined as P<0.05.

Results

In our study, the mean age of patients in the three groups was comparable (P>0.05), i.e., group IB was 39.23±11.78 years, group HB was 43.63±11.33 years, and group IL was 39.8±12.07 years. The time of onset of sensory block was 6.57±1.794 minutes in group IB, 2.30±1.343 minutes in group HB, and 4.57±1.960 minutes in group IL, and this variation was statistically highly significant (P<0.001). A total of 15 patients suffered hypotension intraoperatively, of which eight belonged to group HB, four to group IB, and the rest to group IL. Intraoperative or postoperative nausea/vomiting was seen in five patients in group IB, two patients in group HB, and one patient in group IL. In the postoperative period, the mean heart rate (HR) was 77.47±4.88/minute in group IB, 68.78±7.88/minute in group HB, and 72.15±8.83/minute in group IL. The data was statistically highly significant (P<0.001).

Conclusion

Our study revealed that 15 mg of isobaric levobupivacaine (3 mL of 0.5%), the new racemic isomer of bupivacaine, was intermediate in its anesthetic properties when compared to isobaric bupivacaine and hyperbaric bupivacaine. The onset of sensory and motor blockade is slower than hyperbaric bupivacaine but faster than isobaric bupivacaine with a higher level of maximum sensory block.

## Introduction

Spinal anesthesia, defined as regional anesthesia obtained by blocking nerves in the subarachnoid space, is a popular and common technique used worldwide for more than a century. Many surgical procedures have been accomplished with this option due to the benefits of an awake patient, ease of placement, rapid onset of action, low drug cost, low stress response, relatively fewer side effects, and short patient turnover [1].

Bupivacaine (1-butyl-2',6'-pipecoloxylidide), a pipecoloxylidide derivative, synthesized in 1957 and introduced in clinical practice in 1963, is widely used. Bupivacaine is a racemic mixture of dextro (D)-isomer and levo (L)-isomer. The dextro-isomer of bupivacaine is more cardiotoxic as compared to the levo-isomer. In 1979, a study reported an increased incidence of bupivacaine and cardiac arrest during regional anesthesia [2-4]. An important aspect of this toxicity is that it involves a significant degree of stereospecificity with the S-isomer showing significantly less cardiac depression effect than the R-isomer [5,6].

Because of bupivacaine’s high affinity for the binding site of plasma proteins, it has the peculiar characteristic of not eliciting clinical signs of drug accumulating in plasma before a relatively advanced stage. The free concentration of the drug in plasma remains low until all the protein binding sites are fully occupied, after which it increases rapidly and toxicity can occur without patients exhibiting signs of central nervous system (CNS) toxicity in awake patients [2,7,8].

These findings generated the search for an alternative to bupivacaine concentrating on amide-linked agents, which in current practice have largely replaced ester-type drugs. Levobupivacaine is an amide local anesthetic that is the isolated S (-) enantiomer of racemic bupivacaine. Levobupivacaine has less cardiotoxic and central nervous system effects in comparison with both R (+) bupivacaine and bupivacaine [9]. Levobupivacaine appears to be a reasonable alternative for racemic bupivacaine in light of lesser cardiotoxicity. Clinical studies comparing levobupivacaine and racemic bupivacaine in epidural and infiltration anesthesia show that both are equally effective [10,11]. Levobupivacaine is a regional anesthetic that is clinically well tolerated in a variety of regional anesthesia procedures, both after bolus administration and continuous postoperative infusion.

Levobupivacaine toxicity reports are rare, and when they do occur, such symptoms are frequently treatable with minimal morbidity and mortality. However, levobupivacaine has not entirely replaced bupivacaine in clinical practice [12]. Moreover, the experience of intrathecal anesthesia with levobupivacaine is not as well documented. Hence, the purpose of this study is to assess the quality and duration of sensory and motor blockade of levobupivacaine and its toxic side effects, if any, compared to intrathecal bupivacaine during infraumbilical surgeries.

## Materials and methods

After approval by the Institutional Ethical Committee (KVG/IEC/12/124) of Kurunji Venkatramana Gowda (KVG) Medical College and Hospital, Sullia, 90 patients aged between 18 and 65 years of either sex with American Society of Anesthesiologists (ASA) physical status I-II who were scheduled for elective abdominoperineal, urological, or lower limb surgeries (short duration) under intrathecal anesthesia were enrolled in this prospective, double-blind, randomized comparative study with written informed consent from January 2013 to June 2014. Patients with medical complications (uncontrolled hypertension, ischemic heart disease (IHD), valvular diseases, hypovolemia, septicemia, and coagulation disorders or on anticoagulant therapy), local infection at the site of the proposed puncture for spinal anesthesia, pregnancy, psychiatric disorders, height < 145 cm, morbid obesity (weight > 130 kg), and known case of hypersensitivity to the amide group of local anesthetics were excluded from the study [12].

The selected patients were randomly allocated into three groups of 30 each by a random number table, prepared by another anesthetist outside the operating room, namely, group HB (3 mL of 0.5% hyperbaric bupivacaine), group IB (3 mL of 0.5% isobaric bupivacaine), and group IL (3 mL of 0.5% isobaric levobupivacaine). Preoperative and operative standards were followed as per hospital guidelines [12].

Intervention

With the patient in the lateral decubitus position, intrathecal anesthesia was performed under aseptic conditions and after local infiltration of the skin with 2% lidocaine. Using 25 G Quincke’s needle with a midline approach at L4-L5, the subarachnoid space was entered (determined by palpation of bony landmarks) with bevel pointing cephalad. The spinal block was changed to L3-L4 if the L4-L5 space was not appreciated. Patients were excluded from the study in case of failure of intrathecal anesthesia, and the case was converted into general anesthesia. Drugs were injected slowly over 10 seconds without barbotage technique and after noting the free flow of cerebrospinal fluid (CSF). The patient was turned supine immediately after the injection with a pillow under their head and put in a neutral position. Thereafter, hemodynamic changes, which include pulse rate, systolic blood pressure (SBP), diastolic blood pressure (DBP), mean arterial pressure (MAP), and peripheral oxygen saturation (SpO_2_), were recorded every two minutes for the first 20 minutes, five minutes for the next 30 minutes, and every 10 minutes thereafter until the end of surgery [12].

Assessment of the quality of anesthesia

Assessment of sensory blockade was tested for pain by pinprick test using a hypodermic needle and for temperature using cold swabs on each side of the midclavicular line, and the time of onset, highest level of sensory blockade, time for two-segment regression of sensory level, and duration of sensory block were noted. This test was done every two minutes for the first 20 minutes, five minutes for the next 30 minutes, every 10 minutes thereafter until the end of the surgery, and then every 30 minutes postoperatively until sensory variables became normal. Motor blockade was assessed using the modified Bromage scale, and the time of onset, degree of motor blockade, and duration of motor blockade were recorded. Both tests were done every two minutes for the first 20 minutes, five minutes for the next 30 minutes, every 10 minutes thereafter until the end of the surgery, and then every 30 minutes postoperatively until motor and sensory variables became normal [12].

Postoperatively, the quality of analgesia was evaluated for pain using the visual analog scale (VAS) and was assessed every 30 minutes until VAS > 4, and supplementary analgesia was given at VAS > 4. Rescue analgesics consisted of intravascular injection of diclofenac sodium 75 mg and repeated after 12 hours if needed with a maximum daily dose of 150 mg [12].

Patients were followed up for six hours in the postoperative ward or in the recovery room. Occurrence of nausea and vomiting, shivering, hypoxia (SpO_2_ < 90%), dry mouth, bradycardia, hypotension, or respiratory depression (respiratory rate (RR) < 8/minute) was recorded to know undesirable side effects. The incidence of hypotension (arterial blood pressure (BP) < 20% of baseline or MAP < 60 mmHg) was treated with injection ephedrine 6 mg IV increments, and bradycardia (heart rate (HR) < 50 beats/minute) was treated with injection atropine 0.6 mg IV stat. Nausea and vomiting were treated with injection ondansetron 4 mg IV. Shivering was treated with warm drapes and warm intravenous fluids [12].

Statistical analysis

Data were collected using a pre-approved proforma and tabulated using the Microsoft Office® Excel software (Microsoft Corp., Redmond, WA, USA). The Statistical Package for the Social Sciences (SPSS) version 24 software for Windows (IBM SPSS Statistics, Armonk, NY, USA) was used for carrying out the statistical analysis. Mean and standard deviation (mean±SD) were used to reflect quantitative variables, whereas frequency and percentage were used to reflect qualitative variables (including age, weight, height, body mass index (BMI), and ASA physical status). We analyzed the time of onset, spread to the maximum level, two-segment regression, and duration of either motor or sensory blockade using a one-way analysis of variance (ANOVA) test with correction according to Bonferroni. The surgical time and hemodynamic variables such as heart rate, mean arterial pressure, systolic blood pressure, and diastolic blood pressure were analyzed using a two-way ANOVA test. Intergroup comparison was done using Tukey’s post hoc test. The incidence of adverse effects was analyzed using a chi-squared test. The analysis was considered significant when the P value was less than 0.05.

## Results

In our study, the mean age of patients in the three groups was comparable (P>0.05), i.e., group IB was 39.23±11.78 years, group HB was 43.63±11.33 years, and group IL was 39.8±12.07 years. There was an almost equal number of cases of both sexes in our study (44 males and 46 females). Also, gender distribution was comparable in three groups (P>0.05) as group IB had 17 females and 13 males, group HB had 16 males and 14 females, and group IL has 15 females and 15 males. Again, the mean height of patients in the three groups was comparable (P>0.05), i.e., 1.67±0.08 m in group IB, 1.61±0.06 m in group HB, and 1.64±0.08 m in group IL. The mean weight of patients in the three groups was also comparable (P>0.05), i.e., 59.67±5.82 kg in group IB, 60.30±9.24 kg in group HB, and 59.13±8.52 kg in group IL.

In our study, more than two-thirds of patients (83.3%) were of ASA grade I, whereas the rest (16.7%) were of ASA grade II, and the distribution of patients according to ASA grade in the three groups was statistically not significant (P>0.05). Among planned surgeries, the majority of cases were operated by surgeons (61.1%), followed by gynecologists (20%), and then by orthopedics (18.9%). Most of the surgeries were completed within 31-60 minutes, whereas three surgeries lasted for 121-150 minutes. Only the intergroup comparison of the duration of surgery between group HB and group IL was statistically significant (P<0.05). In about one-third of cases, the highest level of anesthesia was T4 (31.1%), followed by T6 (22.2%). Anesthesia reached up to T7 in four cases, whereas it reached the highest up to T2 in five cases in group IL (Table 1).

**Table 1:** Anesthetics and surgical characteristics of the three groups of subjects. HB: hyperbaric bupivacaine group, IB: isobaric bupivacaine group, IL: isobaric levobupivacaine group, SD: standard deviation, ASA: American Society of Anesthesiologists, OBG: obstetrician-gynecologist

Variable	Number (%)/mean±SD			P value
	IB (n=30)	HB (n=30)	IL (n=30)	
ASA grade				
Grade I (n=75)	28 (93.3)	22 (73.3)	25 (83.3)	>0.05
Grade II (n=15)	2 (6.7)	8 (26.7)	5 (16.7)	
Type of surgery				
OBG (n=18)	3 (10)	8 (26.7)	7 (23.3)	>0.05
General surgery (n=55)	19 (63.3)	18 (60)	18 (60)	
Orthopedics (n=17)	8 (26.7)	4 (13.3)	5 (16.7)	
Duration of surgery (minutes)	64.50±29.80	71.43±35.81	51.67±20.52	<0.05
Highest dermatome level				
T2 (n=5)	0 (0)	0 (0)	5(16.7)	<0.001
T4 (n=28)	3 (10)	17 (56.7)	8 (26.7)	
T5 (n=10)	3 (10)	4 (13.3)	3 (10)	
T6 (n=20)	4 (13.3)	8 (26.7)	8 (26.7)	
T7 (n=4)	0 (0)	1 (3.3)	3 (10)	
T8 (n=10)	8 (26.7)	0 (0)	2 (6.7)	
T10 (n=13)	12 (40)	0 (0)	1 (3.3)	

The time of the onset of sensory block was 6.57±1.794 minutes in group IB, 2.30±1.343 minutes in group HB, and 4.57±1.960 minutes in group IL, and this variation was statistically highly significant (P<0.001). The mean time for the two-segment regression of sensory blockade was the highest in group HB (114.13±20.068 minutes), whereas it was 97.13±9.677 minutes in group IB and 95.53±22.106 minutes in group IL, and this variation was statistically highly significant (P<0.001) (Table 2).

**Table 2:** Comparison between sensory and motor blockade among the three groups of subjects using the ANOVA test. SD: standard deviation, SE: standard error, confidence interval: CI, UL: upper limit, LL: lower limit, Min: minimum value, Max: maximum value, HB: hyperbaric bupivacaine group, IB: isobaric bupivacaine group, IL: isobaric levobupivacaine group, ANOVA: analysis of variance

Group	Number	Mean	SD	SE	95% CI for mean		Min	Max	F	P value
					LL	UP				
Time of the onset of sensory blockade to reach T10 (minutes)										
IB	30	6.57	1.794	0.328	5.90	7.24	4	11	46.274	<0.001
HB	30	2.30	1.343	0.245	1.80	2.80	0	6		
IL	30	4.57	1.960	0.358	3.83	5.30	2	10		
Time for the maximum level of sensory blockade (minutes)										
IB	30	8.07	1.413	0.258	7.54	8.59	6	11	22.402	<0.001
HB	30	5.53	1.943	0.355	4.81	6.26	2	10		
IL	30	8.83	2.493	0.455	7.90	9.76	4	14		
Time for the two-segment regression of sensory blockade (minutes)										
IB	30	97.13	9.677	1.767	93.52	100.75	74	123	9.708	<0.001
HB	30	114.13	20.068	3.664	106.64	121.63	80	165		
IL	30	95.53	22.106	4.036	87.28	103.79	60	150		
Duration of sensory blockade (minutes)										
IB	30	205.10	18.129	3.310	198.33	211.87	174	234	16.717	<0.001
HB	30	260.60	43.481	7.938	244.36	276.84	180	335		
IL	30	231.47	43.933	8.021	215.06	247.87	180	360		
Time for the onset of motor blockade (minutes)										
IB	30	11.77	3.857	0.704	10.33	13.21	5	21	36.894	<0.001
HB	30	5.57	1.995	0.364	4.82	6.31	2	10		
IL	30	7.17	2.534	0.463	6.22	8.11	5	11		
Duration of motor blockade (minutes)										
IB	30	209.90	13.548	2.473	204.84	214.96	180	230	36.894	<0.001
HB	30	248.97	42.306	7.724	233.17	264.76	150	350		
IL	30	240.23	39.113	7.141	225.63	254.84	165	360		
Timing of rescue analgesia (minutes)										
IB	25	223.64	24.61	16.007	153.63	219.11	190	300	0.359	0.699
HB	22	287.73	64.51	25.666	158.51	263.49	120	380		
IL	24	254.50	48.85	20.505	161.66	245.54	150	360		

Intergroup comparison for the time of the onset of sensory blockade among all three groups was significant (P<0.05). Intergroup comparison for the time for the maximum level of sensory blockade between IB and HB, and IL and HB was statistically significant (P<0.05), whereas there was no statistically significant difference between group IL and group IB (Table 3).

**Table 3:** Comparison between sensory and motor blockade among the three groups of subjects using Student’s t-test. *The mean difference is significant at the 0.05 level. IB: isobaric bupivacaine group, IL: isobaric levobupivacaine group, HB: hyperbaric bupivacaine group

Groups	Mean difference	P value	95% Confidence interval	
			Lower limit	Upper limit
Time of the onset of sensory blockade to reach T10 (minutes)				
IB-HB	4.27(*)	<0.001	3.18	5.35
IB-IL	2.00(*)	<0.001	0.92	3.08
IL-HB	2.27(*)	<0.001	1.18	3.35
Time for the maximum level of sensory blockade (minutes)				
IB-HB	2.53(*)	<0.001	1.27	3.79
IL-IB	0.77	0.423	-0.49	2.03
IL-HB	3.30(*)	<0.001	2.04	4.56
Time for the two-segment regression of sensory blockade (minutes)				
IB-IL	1.60	1.000	-9.82	13.02
HB-IB	17.00(*)	0.001	5.58	28.42
HB-IL	18.60(*)	<0.001	7.18	30.02
Duration of sensory blockade (minutes)				
HB-IB	55.50(*)	<0.001	32.06	78.94
HB-IL	29.13(*)	0.010	5.69	52.57
IL-IB	26.37(*)	0.022	2.93	49.81
Time for the onset of motor blockade (minutes)				
IB-HB	6.20(*)	<0.001	4.37	8.03
IB-IL	4.60(*)	<0.001	2.77	6.43
IL-HB	1.60	0.107	-0.23	3.43
Duration of motor blockade (minutes)				
HB-IB	39.07(*)	<0.001	17.53	60.61
HB-IL	8.73	0.975	-12.81	30.27
IL-IB	30.33(*)	0.003	8.79	51.87
Timing of rescue analgesia (minutes)				
HB-IB	64.09	1.000	-48.20	97.47
HB-IL	33.23	1.000	-65.44	80.24
IL-IB	30.86	1.000	-55.60	90.07

**Figure 1:**
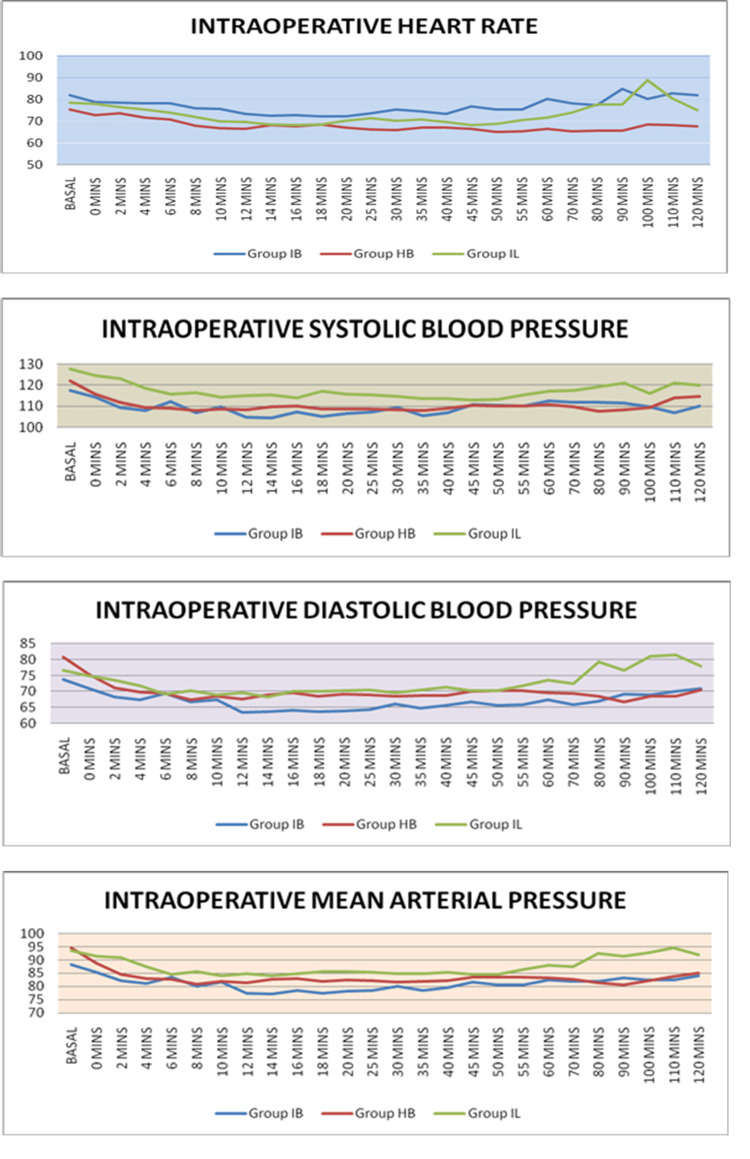
Comparison of intraoperative mean hemodynamic readings among the three groups of subjects using the ANOVA test. A: Intraoperative mean heart rate. B: Intraoperative systolic blood pressure. C: Intraoperative diastolic blood pressure. D: Intraoperative mean arterial pressure. ANOVA: analysis of variance

In group IB, the mean heart rate was 84.87±72.16/minute. In group HB, it was 75.16±65.08/minute. In group IL, it was 88.66±68.10/minute. The variation in the mean heart rate from six to 12 minutes and from 25 to 100 minutes was statistically significant. Intergroup comparison between group IB and group HB was most significant starting from six minutes to 100 minutes. Comparison between group IB and group IL was significant at 45-60 minutes and between group HB and group IL was significant at 70-100 minutes. The variation in the mean SBP was statistically significant at 0, 2, 4, 8, 12, 14, 18, 20, 25, 35, 80, and 90 minutes, with the most significant variation observed at two-minute time intervals (P<0.001). Intergroup comparison between group IL and group IB was significant from the start to 40 minutes, except at six and 10 minutes. Comparison between group IL and group HB was significant only at 0, 2, 4, 8, 18, 80, and 90 minutes. In these three groups, about eight patients in group HB, three patients in group IB, and three patients in group IL had hypotension. Intergroup comparison for mean DBP between group IL and group IB was significant at 18, 20, 25, and 80 minutes. Between group IL and group HB, it was significant at 80 and 90 minutes. This variation in MAP was statistically significant at time intervals of 2, 12, 14, 16, 18, 20, 25, 35, 80, and 90 minutes. Intergroup comparison between group IB and group IL was significant at 2, 12, 14, 16, 18, 20, 25, 35, 40, and 80 minutes. Comparison between group HB and group IL was significant at 80 and 90 minutes (Figure 1).

**Figure 2:**
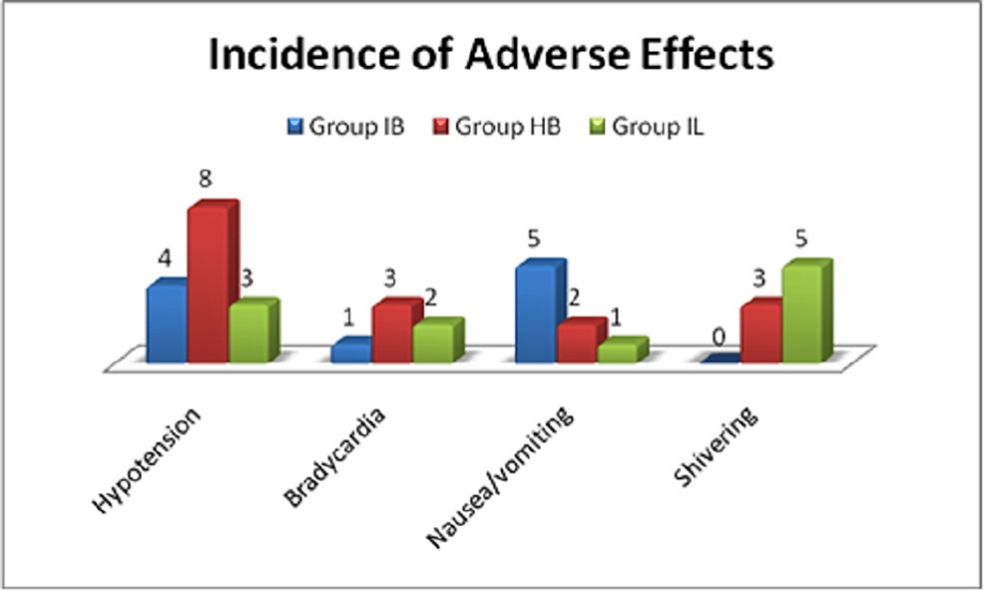
Incidence of adverse effects among the three groups of subjects.

A total of 15 patients suffered hypotension intraoperatively, of which eight belonged to group HB, four to group IB, and the rest to group IL. Intraoperative or postoperative nausea/vomiting was seen in five patients in group IB, two patients in group HB, and one patient in group IL. Shivering was seen in five patients administered with isobaric levobupivacaine and three patients administered with hyperbaric bupivacaine. The least common complication was bradycardia, which was seen in 10% of the patients in group HB, 6.7% of the patient in group IL, and 3.3% of the patients in group IB. However, this difference in the occurrence of adverse events among the three groups was not statistically significant (Figure 2).

In the postoperative period, the mean heart rate was 77.47±4.88/minute in group IB, 68.78±7.88/minute in group HB, and 72.15±8.83/minute in group IL, and statistically, this difference was highly significant (P<0.001). The mean SBP was 112.77±7.80 mmHg in group IB, 115.80±9.05 mmHg in group HB, and 114.24±9.00 mmHg in group IL. The mean DBP was 69.02±5.09 mmHg in group IB, 72.56±7.25 mmHg in group HB, and 71.66±6.77 mmHg in group IL. The mean MAP was 83.60±4.69 mmHg in group IB, 86.98±7.38 mmHg in group HB, and 85.86±7.12 mmHg in group IL, and this difference had no statistical significance (Table 4).

**Table 4:** Comparison of postoperative mean hemodynamic readings among the three groups of subjects using the ANOVA test. SD: standard deviation, SE: standard error, CI: confidence interval, UL: upper limit, LL: lower limit, Min: minimum value, Max: maximum value, HB: hyperbaric bupivacaine group, IB: isobaric bupivacaine group, IL: isobaric levobupivacaine group, ANOVA: analysis of variance

Group	Number	Mean	SD	SE	95% CI for mean		Min	Max	F	P value
					LL	UL				
Mean heart rate (beats/minute)										
IB	30	77.47	4.88	0.89	75.65	79.30	68.38	89.46	10.541	<0.001
HB	30	68.78	7.88	1.44	65.83	71.72	55.69	84.08		
IL	30	72.15	8.83	1.61	68.85	75.45	54.31	90.38		
Mean systolic blood pressure (mmHg)										
IB	30	112.77	7.80	1.42	109.85	115.68	101.31	130.85	0.922	0.402
HB	30	115.80	9.05	1.65	112.42	119.17	99.92	137.77		
IL	30	114.24	9.00	1.64	110.88	117.61	95.00	138.23		
Mean diastolic blood pressure (mmHg)										
IB	30	69.02	5.09	0.93	67.12	70.92	60.77	81.00	2.454	0.092
HB	30	72.56	7.25	1.32	69.86	75.27	57.23	87.23		
IL	30	71.66	6.77	1.23	69.13	74.19	60.08	83.08		
Mean of mean arterial pressure (mmHg)										
IB	30	83.60	4.69	0.85	81.85	85.35	75.62	95.10	2.090	0.130
HB	30	86.98	7.38	1.34	84.22	89.74	71.46	104.08		
IL	30	85.86	7.12	1.30	83.19	88.52	72.54	96.79		

The intergroup comparison for mean heart rate showed a statistical signiﬁcance between group IB and both groups HB and IL (P<0.05). However, intergroup comparison (IB-HB, IB-IL, and IL-IB) for mean systolic blood pressure, mean diastolic blood pressure, and mean arterial blood pressure was found to be statistically non-signiﬁcant (P>0.05) (Table 5).

**Table 5:** Comparison of postoperative mean hemodynamic readings among the three groups of subjects using Student’s t-test. *The mean difference is significant at the 0.05 level. IB: isobaric bupivacaine group, IL: isobaric levobupivacaine group, HB: hyperbaric bupivacaine group

Pair	Mean difference	P value	95% confidence interval	
			Lower bound	Upper bound
Mean heart rate (beats/minute)				
IB-HB	8.6949(*)	<0.001	4.0334	13.3564
IB-IL	5.3231(*)	0.020	0.6616	9.9846
IL-HB	3.3718	0.243	-1.2897	8.0333
Mean systolic blood pressure (mmHg)				
HB-IB	3.0282	0.534	-2.4171	8.4735
HB-IL	1.5513	1.000	-3.8940	6.9966
IL-IB	1.4769	1.000	-3.9684	6.9222
Mean diastolic blood pressure (mmHg)				
HB-IB	3.5462	0.108	-0.5153	7.6076
HB-IL	0.9026	1.000	-3.1589	4.9641
IL-IB	2.6436	0.347	-1.4179	6.7051
Mean of mean arterial pressure (mmHg)				
HB-IB	3.3778	0.143	-0.7296	7.4851
HB-IL	1.1231	1.000	-2.9843	5.2304
IL-IB	2.2547	0.551	-1.8527	6.3621

## Discussion

In this study, we compared the anesthetic properties of levobupivacaine with those of bupivacaine and also the incidence of adverse effects associated with their use. We conducted our study with a dose of 3 mL of 0.5% of each anesthetic, and our study findings were comparable with several studies [13-20] using the same dose, except for Glaser et al. [21], who used a dose of 3.5 mL, and Cuvas et al. [22] and Vanna et al. [23], who both used a dose of 2.5 mL.

Most of the studies have demonstrated that hyperbaric bupivacaine has a faster onset of action (sensory blockade) compared to isobaric levobupivacaine, which in turn is faster than isobaric bupivacaine. Our study also yielded similar results (IB: 6.57±1.79 minutes, HB: 2.30±1.34 minutes, IL: 4.57±1.96 minutes) [13-20]. However, our time of onset matched only that of Gulec et al. (2.81±0.66 minutes) [19] for hyperbaric bupivacaine, Mehta et al. (4.38±1.53 minutes) [14] for isobaric levobupivacaine, and Raikwar et al. (6.36±1.38minutes) [20] for isobaric bupivacaine.

We found that time for sensory blockade of the highest dermatome took less time for hyperbaric bupivacaine, whereas it was comparable between isobaric bupivacaine and isobaric levobupivacaine (IB: 8.07±1.41 minutes, HB: 5.53±1.94 minutes, IL: 8.83±2.49 minutes). Our results are in contrast with the findings of D’Souza et al. (HB: 4.5 minutes, IL: 5.5 minutes) [18] and Gulec et al. (HB: 7.79±1.44 minutes, IL: 7.68±1.89 minutes) [19], who concluded that the time taken for the maximum sensory blockade is comparable between hyperbaric bupivacaine and isobaric levobupivacaine.

In our study, the time taken for the anesthetic to regress two dermatome levels was comparable between isobaric bupivacaine and isobaric levobupivacaine but was more in hyperbaric bupivacaine (IB: 97.13±9.67 minutes, HB: 114.13±20.06 minutes, IL: 95.53±22.10 minutes). These findings are in agreement with the findings of Glaser et al. [21], Vanna et al. [23], Cuvas et al. [22], and Mantouvalou et al. [16], but Gulec et al. [19] found that hyperbaric bupivacaine regressed faster than isobaric bupivacaine (HB: 76.28±7.16 minutes, IL: 82.19±6.05 minutes).

In our study, hyperbaric bupivacaine provided long-lasting sensory anesthesia, followed by isobaric levobupivacaine, and then by isobaric bupivacaine (IB: 205.10±18.12 minutes, HB: 260.60±43.48 minutes, IL: 231.47±43.93 minutes). The findings of our study are consistent with those of Glaser et al. (IB: 237±88 minutes, IL: 228±77 minutes) [21], Fattorini et al. (IB: 381±105 minutes, IL: 391±96 minutes) [13], Mehta et al. (IB: 175.76±50 minutes, IL: 189.4±42.9 minutes) [14], and Sahin et al. (HB: 259.65 minutes, IL: 245.15 minutes) [17], whereas other findings were opposite of ours [15,16,18,22,23].

Limited studies have reported the incidence of adverse effects, and those were compared with the adverse effects reported in our study. In our study, hyperbaric bupivacaine had a high incidence of hypotension, which was similar to the findings published in the study by Vanna et al. [23]. However, in our study, the incidence of bradycardia differed from the study by Vanna et al. [23]. The studies by Vanna et al. [23] and Cuvas et al. [22] are the only studies that reported shivering. Vanna et al. [23] reported maximum cases of shivering with isobaric levobupivacaine as in our study, but Cuvas et al. [22] in stark contrast did not report any cases of shivering from the isobaric levobupivacaine group. with respect to the incidence of hypotension and bradycardia with isobaric bupivacaine, our study findings are similar to those of Cuvas et al. [22], Mantouvalou et al. [16], and Raikwar et al. [20] but the complete opposite of those of Sathitkarnmanee et al. [15]. Nausea and vomiting were noted in all studies with comparable frequencies, but in our study, isobaric bupivacaine had the highest incidence of nausea and vomiting, which is in agreement with the findings of Cuvas et al. [22].

In our study, we observed that intraoperative hemodynamic parameters were better in the group that received isobaric levobupivacaine in comparison to the groups that received isobaric bupivacaine and hyperbaric bupivacaine. Solakovic [24] observed that the isobaric version of bupivacaine had better hemodynamic stability compared to the hyperbaric version, which was in contrast to our observations. Dimarzio et al. [25] noted better hemodynamic stability with isobaric levobupivacaine in comparison to hyperbaric bupivacaine just as noted by us.

Strength and limitations

The prospective nature and the inclusion of three comparative groups were the strength of the study, but we realized the single-centric nature of the study as the limitation, so we suggest future studies expand to multicentric studies for better generalizability of the present study findings.

## Conclusions

Our study revealed that 15 mg of isobaric levobupivacaine (3mL of 0.5%), the levo-isomer of bupivacaine, was intermediate in its anesthetic properties when compared to isobaric bupivacaine and hyperbaric bupivacaine. The onset of sensory and motor blockade is slower than hyperbaric bupivacaine but faster than isobaric bupivacaine with a higher level of maximum sensory block. It also has the advantages of predictable onset and consistent performance. The duration of sensory and motor blockade was shorter compared to hyperbaric bupivacaine, thus offering early mobility and thus can be preferred in daycare surgeries. With the advantage of minimum cardiotoxicity and predictable and consistent performance with better hemodynamic stability, 0.5% isobaric levobupivacaine can be a better alternative to 0.5% hyperbaric bupivacaine and 0.5% isobaric bupivacaine for lower limb or abdominoperineal surgeries, where early recovery is well appreciated by the patients due to early ambulation and faster home discharge.

## References

[1] Barasch PG, Collen BF (2006). Clinical anesthesia, 6th edition.

[2] Albright GA (1979). Cardiac arrest following regional anesthesia with etidocaine or bupivacaine. Anesthesiology.

[3] Moller RA, Covino BG (1988). Cardiac electrophysiologic effects of lidocaine and bupivacaine. Anesth Analg.

[4] Berde CB, Strichartz GR (2010). Local anesthetics. Miller's anesthesia, 7th edition.

[5] Whiteside JB, Wildsmith JA (2001). Developments in local anaesthetic drugs. Br J Anaesth.

[6] Feldman HS, Arthur GR, Pitkanen M, Hurley R, Doucette AM, Covino BG (1991). Treatment of acute systemic toxicity after the rapid intravenous injection of ropivacaine and bupivacaine in the conscious dog. Anesth Analg.

[7] Friedman GA, Rowlingson JC, DiFazio CA, Donegan MF (1982). Evaluation of the analgesic effect and urinary excretion of systemic bupivacaine in man. Anesth Analg.

[8] Yamashiro H (1977). Bupivacaine-induced seizure after accidental intravenous injection, a complication of epidural anesthesia. Anesthesiology.

[9] McLeod GA, Burke D (2001). Levobupivacaine. Anaesthesia.

[10] Cox CR, Faccenda KA, Gilhooly C, Bannister J, Scott NB, Morrison LM (1998). Extradural S(-)-bupivacaine: comparison with racemic RS-bupivacaine. Br J Anaesth.

[11] Lyons G, Columb M, Wilson RC, Johnson RV (1998). Epidural pain relief in labour: potencies of levobupivacaine and racemic bupivacaine. Br J Anaesth.

[12] Burlacu CL, Buggy DJ (2022). Update on local anesthetics: focus on levobupivacaine. Ther Clin Risk Manag.

[13] Fattorini F, Ricci Z, Rocco A, Romano R, Pascarella MA, Pinto G (2006). Levobupivacaine versus racemic bupivacaine for spinal anaesthesia in orthopaedic major surgery. Minerva Anestesiol.

[14] Mehta A, Gupta V, Wakhloo R (2007). Comparative evaluation of intrathecal administration of newer local anesthetic agents ropivacaine and levobupivacaine with bupivacaine in patients undergoing lower limb surgery. Internet J Anesthesiol.

[15] Sathitkarnmanee T, Thongrong C, Tribuddharat S, Bn MT, Bn KP, Bn RK (2011). A comparison of spinal isobaric levobupivacaine and racemic bupivacaine for lower abdominal and lower extremity surgery. J Med Assoc Thai.

[16] Mantouvalou M, Ralli S, Arnaoutoglou H, Tziris G, Papadopoulos G (2008). Spinal anesthesia: comparison of plain ropivacaine, bupivacaine and levobupivacaine for lower abdominal surgery. Acta Anaesthesiol Belg.

[17] Sahin SH, Inal M, Alagol A (2011). Effects of bupivacaine versus levobupivacaine on pulmonary function in patients with chronic obstructive pulmonary disease undergoing urologic surgery: a randomized, double-blind, controlled trial. Curr Ther Res Clin Exp.

[18] D’Souza AD, Saldanha NM, Monteiro AD, Harshavardhan H (2014). Comparison of Intrathecal hyperbaric 0.5% Bupivacaine, isobaric 0.5% Levobupivacaine and isobaric 0.75% Ropivacaine for lower abdominal surgeries. Int J Health Sci Res.

[19] Gulec D, Karsli B, Ertugrul F, Bigat Z, Kayacan N (2014). Intrathecal bupivacaine or levobupivacaine: which should be used for elderly patients?. J Int Med Res.

[20] Raikwar S, Gupta P, Thakur R (2014). Comparative evaluation of intrathecal administration of ropivacaine, levobupivacaine and bupivacaine in patients undergoing lower limb surgeries. J Evolution Med Dental Sci.

[21] Glaser C, Marhofer P, Zimpfer G, Heinz MT, Sitzwohl C, Kapral S, Schindler I (2002). Levobupivacaine versus racemic bupivacaine for spinal anesthesia. Anesth Analg.

[22] Cuvas O, Er AE, Ongen E, Basar H (2008). Spinal anesthesia for transurethral resection operations: bupivacaine versus levobupivacaine. Minerva Anestesiol.

[23] Vanna O, Chumsang L, Thongmee S (2006). Levobupivacaine and bupivacaine in spinal anesthesia for transurethral endoscopic surgery. J Med Assoc Thai.

[24] Solakovic N (2010). Comparison of hemodynamic effects of hyperbaric and isobaric bupivacaine in spinal anesthesia. Med Arh.

[25] Dimarzio G, d’Elia A, Ciao A, Vessicchio L, Lettieri B (2021). Comparison between isobaric levobupivacaine and hyperbaric bupivacaine in subarachnoid anesthesia (SAB) for cesarean delivery: our experience. https://www.unisa.it/uploads/5747/02_dimarzio.pdf.

